# Design for recycling in electronic manufacturing: enabling circularity and lower impact manufacturing through heterogeneous integration and lower impact recovery

**DOI:** 10.1038/s44296-026-00098-8

**Published:** 2026-03-16

**Authors:** Tianwei Zhang, Jonathon Harwell, Joseph Cameron, Shoushou Zhang, Hadi Heidari, Jeff Kettle

**Affiliations:** 1 James Watt School of Engineering, University of Glasgow, Glasgow, UK; 2 Bangor College, Central South University of Forestry and Technology, Changsha, China

**Keywords:** Engineering, Environmental sciences, Materials science

## Abstract

The adoption of ‘Design-for-Recycling’ (DfR) approaches for manufacturing Electrical and Electronic Equipment is still in its infancy. However, growing interest in DfR is driven by its potential to address the global challenge of waste from electrical and electronic equipment (WEEE) and reduce the environmental footprint of electronics. In this study, a DfR approach that enables up to 99% material recovery using scalable laboratory methods is presented. Through Life Cycle Assessment (LCA), a 90% reduction in environmental impact compared to conventional Flame-Retardant Level 4 (FR4)-based printed circuit board assemblies manufacturing is demonstrated. To achieve this, a fully additive process is adopted with Heterogeneous integration using Ultra-Precise Deposition (UPD) to provide a more compact form factor with lower material use than conventional approaches. For recycling trials, selective recovery using Iron Chloride (FeCl₃) was adopted, enabling silver (Ag) to be recovered by mechanical filtration. The LCA results indicate a notable reduction in environmental impact and human toxicity, primarily due to the lower substrate footprint, reduced reagent use, the ease of disassembly and resource retention enabled by the DfR design. The results show that the integration of DfR principles with recycling establishes a new standard for electronics manufacturing, where technological performance and environmental accountability are simultaneously pursued.

## Introduction

Electrical and electronic equipment (EEE) has become a vital part of modern life, enabling much of the global population to enjoy improved living standards. However, the current methods of manufacturing, using, and disposing of electronics are fundamentally unsustainable. Only 17% of WEEE, is formally processed^1^ with the majority ending up in landfills, being incinerated, or being handled using substandard methods^2^. A significant portion of WEEE consists of printed circuit boards (PCBs). The most widely used substrate, FR4, accounts for approximately 70% of PCB waste by mass^3,4^. and presents a significant challenge due to its content of toxic substances, which limit recyclability. This leads to the loss of highly purified metals (e.g., copper, silver, nickel), all of which are finite resources with limited reserves. The recovery of metallic and non-metallic resources from waste PCBs is not an effective solution currently due to the lack of specialised facilities, expensive recovery processes, and informal collection of WEEE^5^. However, the processes that are currently used for metal recovery include pyrometallurgy^6^. hydrometallurgy^7^. electrometallurgy^8^ All of which will produce toxic furans and dioxins due to the combustion of brominated flame retardants (BFR) in the FR4 substrate material. In addition, conventional leaching solutions for the recovery of precious metals are usually based on hydrochloric and nitric acid, which are highly corrosive and generate hazardous gases such as nitrogen dioxide^9^.

To address the FR4 recycling issue, flexible thermoset polymers such as polyimide or polyester have been explored as potentially recyclable alternative substrates^10^. However, their high chemical resistance means that recycling remains a challenge^11^. Utilising biodegradable substrate materials for PCBs significantly enhances the environmental credentials of EEE, as they can be derived from renewable resources like agricultural waste and exhibit a reduced carbon footprint^12^. Recent studies have demonstrated the possibility of using flame-retarded PLA or flax composites as a sustainable substitute for traditional substrates, showing reliable performance in complex circuit assemblies and initial degradation in composting environments^13^. However, to be applicable in PCB manufacturing, they must meet specific requirements. The substrate should still be able to withstand temperatures between 120-150 °C without physical deformation. The surface must also be smooth and non-absorbent to water or ink to maintain clarity during circuit printing.

Changing substrates alone will not ensure the sustainability of EEE. This requires a broader assessment of manufacturing operations where sustainability is considered a key decision attribute alongside traditional metrics like cost and quality^14^. Apart from the recycling of WPCBs, the global production of all rigid and FPCBs itself generates 70 thousand tons of copper waste yearly because of the use of subtractive wet etching methods to pattern the tracks^4^. Switching to additive techniques^15,16^. such as using inkjet printing for conductive tracks, can minimise material waste by precisely applying resources where needed. This markedly reduces the consumption of raw materials^17,18^ and hence reduces CO_2_ emissions significantly^19^. However, the sustainability advantage of printed electronics is only fully realised when a design-for-recycling approach is adopted, particularly for recovering valuable materials like silver (Ag) from conductive inks at the end-of-life^20^. Some progress has been made towards recyclable PCBs, such as the work by Khrustalev et al., who used polylactic acid (PLA) and fibreglass substrates with copper foil^21^. However, their approach still relied on subtractive patterning and the use of toxic tetrahydrofuran (THF) for recycling. There is an ongoing need for more sustainable, additive manufacturing strategies that avoid hazardous solvents and support effective end-of-life material separation.

This paper aims to tackle the environmental challenges of PCBs by combining additive manufacturing for efficient material use on biodegradable substrates and adopting a Design-for-Recycling (DfR) approach to ensure that valuable critical raw materials can be easily and economically recovered at the end-of-life (EoL). This aligns with building a circular economy for electronics, encompassing the entire product lifecycle from design to recycling, where a key element involves component disassembly systems enabling reuse and recovery to extend material lifecycles^22,23^. A detailed comparison of commercially available biodegradable substrates is conducted to evaluate their processability. Conductive traces were applied using additive manufacturing, while the Integrated Circuits (ICs) were integrated using an Ultra-Precise Deposition (UPD) system in order to produce high-performance circuit blocks with zero waste. Finally, we were able to fully recover the valuable silver from the conductive tracks using a low impact solvent; dilute ferric chloride (FeCl_3_) solution. This has much lower environmental impact than, e.g., nitric acid and simultaneously enables the debonding of the ICs and surface-mounted components for easy re-use in future devices. This DfR approach can be considered a promising route for realising sustainable electronics manufacture.

## Results

### Substrate selection

We have measured the surface roughness and temperature stability among all the selected substrates. The basic information and results of biodegradable substrate can be found in Supplementary Section ‘Basic Information and Results of Biodegradable Substrates’. All substrates used in this study are shown in Supplementary Fig. 1.

**Fig. 1:**
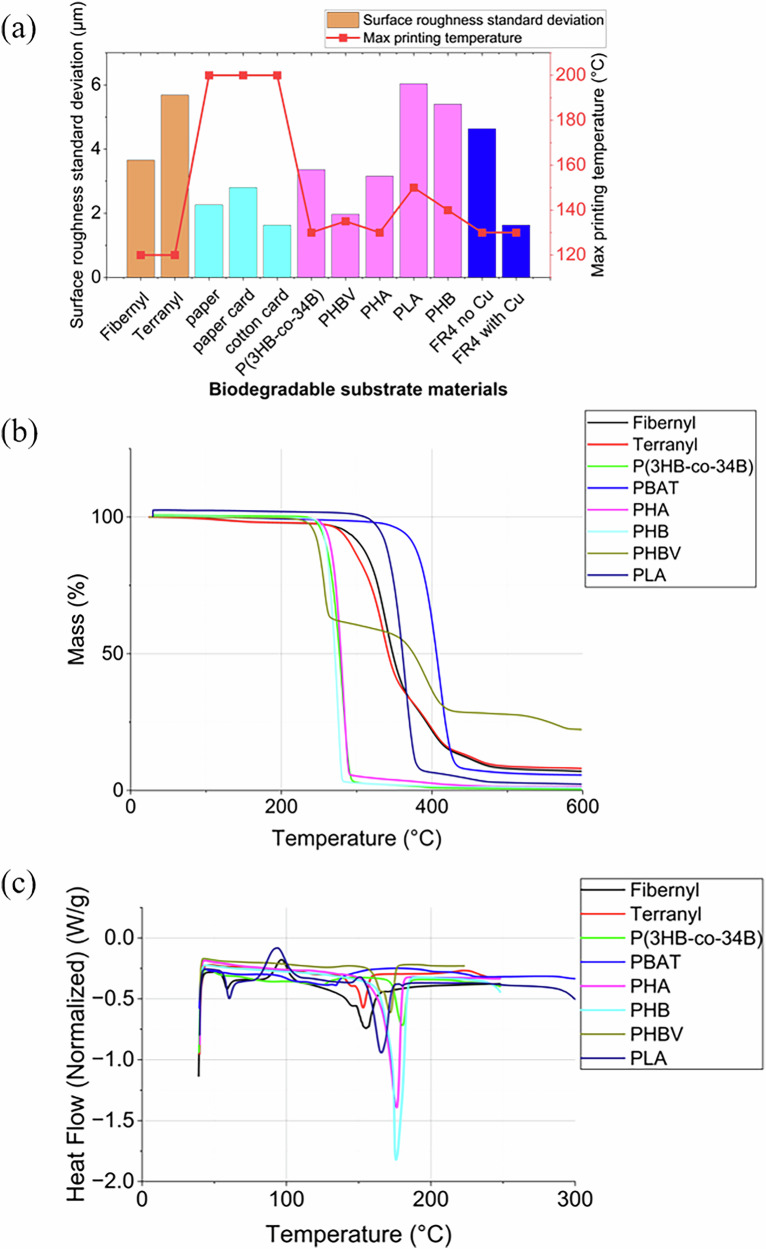
Biodegradable substrate comparison. **a** Comparison of surface roughness, measured by an Alicona optical profilometer; **b** Comparison of thermal stability measured by TGA and **c** Comparison of thermal transitions characterisation by DSC for biodegradable substrates and FR-4 reference materials, (endo negative).

The surface roughness of biodegradable substrates compared to FR-4 and paper was studied to determine their suitability for additive manufacturing of PCBs. Substrates with lower roughness were shown to have clearer, more precise circuit patterns with fewer printing passes and a comparison of surface roughness is shown in Fig. 1. A baseline is given for the reference material, FR-4 without copper (FR-4 no Cu), which exhibits a relatively rough texture. Notably, Terranyl and Fibernyl sheets, both containing fibres in their bioplastic composition, exhibit higher surface roughness levels. These materials also demonstrated the lowest thermal stability among the tested substrates, likely due to the thermal sensitivity of their constituent biopolymers and filler interactions. PLA and PHB display a slightly higher roughness compared to FR-4, but they offer better heat resistance among the tested biodegradable polymers, which is advantageous for processes involving elevated temperatures. On the other hand, P(3HB-co-4HB), Poly (3-hydroxybutyrate- co -3-hydroxyvalerate) (PHBV), and Polyhydroxyalkanoates (PHA) exhibit significantly lower roughness levels, approaching half of that observed in FR-4, making them suitable for finer quality prints. Similarly, standard office paper, along with paper card sheets and cotton card sheets, exhibits a reduction in surface roughness of up to 50% compared to FR-4, while being capable of withstanding temperatures up to 200 °C. This property ensures they remain stable and do not deform during the curing process, which is essential for maintaining the integrity of printed circuits.

The thermogravimetric analysis (TGA) and differential scanning calorimetry (DSC) results for various biodegradable substrates provide insights into their thermal stability and decomposition behaviour. The TGA data indicate that all materials exhibit minimal mass loss below 250 °C. Above 250 °C, significant mass loss occurs, reflecting the breakdown of main polymer chains, with degradation onset temperatures varying due to differing compositional structures.

The DSC results clarify the thermal properties. PLA and the PLA-containing material Fibernyl, exhibit a crystallisation peak at around 90 °C, consistent with previous observation for PLA^24^. Notably, materials like P(3HB-co-4HB) and PHBV remain stable beyond 150 °C, suitable for curing processes at around 120 °C without degradation risks. Noting that the curing process is the stage in the circuit printing process that involves the highest temperatures, it is designed to ensure the complete evaporation of liquids, typically solvents, from the ink. As a result, materials like P(3HB-co-4HB) and PHBV are the most suitable biodegradable substrates for printing based on their better temperature stability. More parameters extracted from DSC and TGA results can be found in Supplementary Table 1. Thanks to the suitable characteristics of P(3HB-co-4HB) and PHBV, so that the die embedding results are great, details can be found in Supplementary Section ‘Die Embedding Result’, including its process flow in Supplementary Fig. 2 and result in Supplementary Fig. 3.

### Printed track performance biodegradable substrates

A critical aspect is the assessment of the electrical conductivity of conductive tracks formed on the chosen substrate. Our work focused on PHBV based on the results in the above section, and several optimisations were made to the printing process and procedure. Conductivity tests were performed on 5 cm lines with a 0.2 mm width and 30 µm thickness (as determined by surface profilometry), as these represent the longest continuous conductive path required for the circuit designs in Section ‘Recycling Trials’. Through optimisation of the printing parameters, including the number of passes, the tracks consistently exhibited resistance values between 2.3 to 2.5 Ω across different substrates, which corresponds to a resistivity of 1.4 × 10^-4^ Ω·cm. This is comparable to a previous report of Ag tracks cured at a similar temperature^25^. These findings highlight that circuit fabrication is possible directly onto biodegradable materials, as low-temperature annealing can be applied, reducing the curl or deformation at higher temperatures. Additionally, the performance of SMD device mounting can be found in the Supplementary Section ‘Packaged/SMD Device Mounting Result’ and especially in Supplementary Fig. 4.

### Additive bonding wire characteristics

**Fig. 2:**
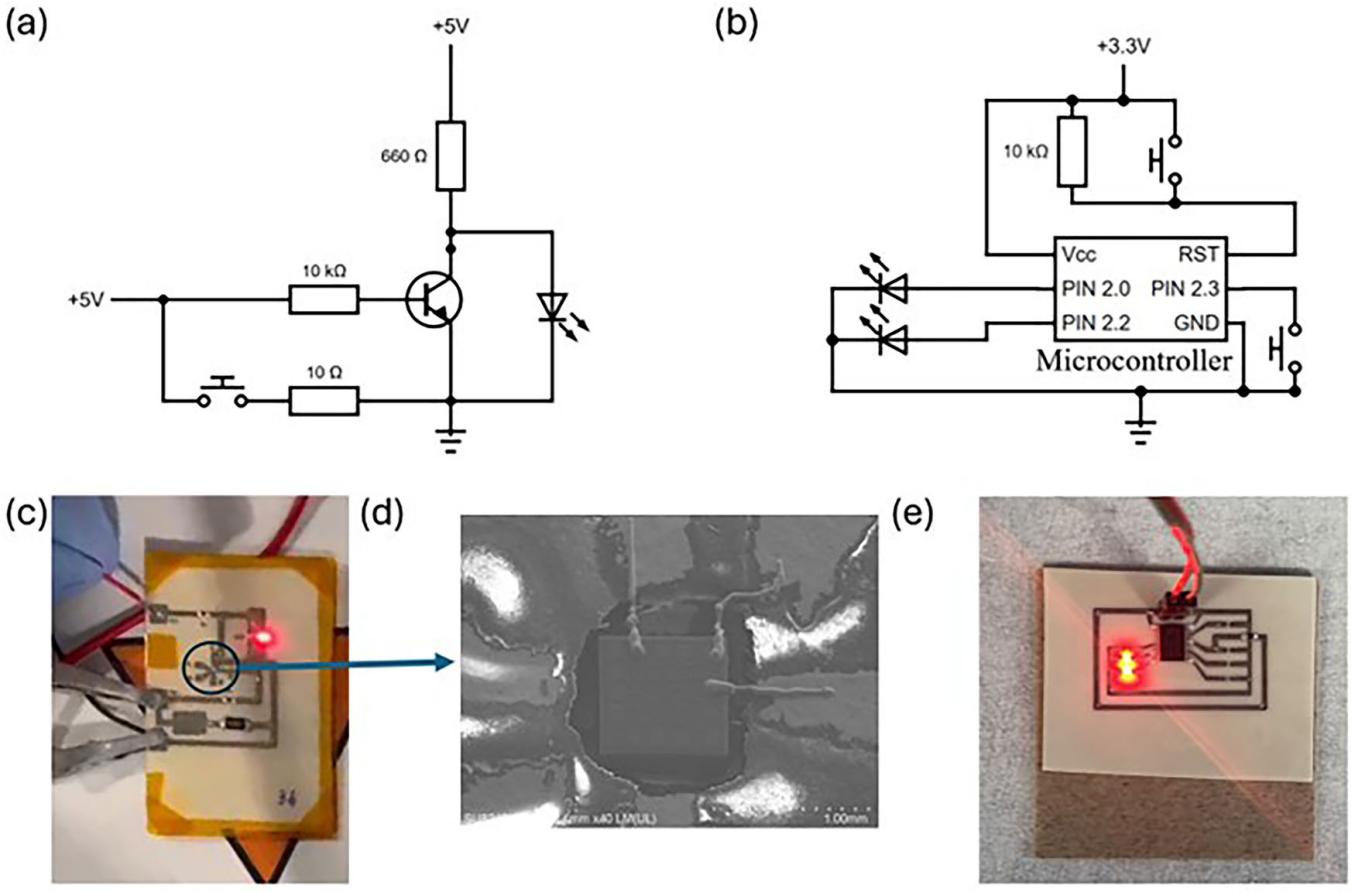
Biodegradable circuit on PHBV. **a** Circuit diagram of IC with BJT array. **b** Circuit diagram of MSP430 microcontroller. **c** Printed BJT circuit on PHBV. **d** SEM view of UPD printed wire bonding to unpackaged IC. **e** Printed MSP430 microcontroller circuit on PHBV.

As depicted in Fig. 2d, UPD printing technology was employed to establish conductive Ag links between the bonding pads of an unpackaged IC containing a Bipolar Junction Transistor (BJT) array and the corresponding circuit tracks. To estimate the resistance of the additive printed wires, a reference wire was printed using a 20 µm diameter nozzle and 9 bar of pressure to create a straight wire bond of 0.5 cm in length. The detailed measurement and calculation steps can be found in Supplementary Section ‘XTPL Resistivity Measurement and Calculation’, and the resistivity (*ρ*) of Ag wire is 3.14 × 10^-6^ Ω·cm. This demonstrates the capability by achieving resistivity values near those of bulk Ag (1.6 × 10^−6^ Ω·cm at 20 °C^26^) and marks a notable improvement from the typical resistivity observed in other printed Ag tracks (~1.4 × 10^−4^ Ω·cm). Compared to traditional Gold wire bonding, which exhibits a resistivity of 46 mΩ per 1 cm segment^27^. The UPD bonding approach offers comparable conductivity with potentially lower material use, contributing to more sustainable and efficient electronic manufacturing processes. Additional properties comparing different wire bonding techniques are presented in Table 1^28^. Importantly, the use of UPD-deposited Ag decreased the thermal resistance of a bond significantly as a result of the higher thermal conductivity of Ag and the lower bond length.

**Table 1:** Properties of different wire bonding techniques

Parameter	Wire bond Au	Wire bond Al-1% Si	UPD Ag
Melting temperature, °C	1064	625	961
Bonding Geometry	25 μm diameter 3.5 mm length	25 μm diameter 3.5 mm length	30 μm diameter 2 mm length
Typical pitch, μm	170 μm perimeter	170 μm perimeter	60 μm perimeter
Lead resistance, mΩ	122	142	32
Lead Inductance, nH	2.6	2.6	1.94
Thermal resistance, °C/mW per bond	56.16	75.11	1.64

### Flexible heterogeneous circuit fabrication

Two demonstrations of circuits were implemented in this paper to demonstrate the approach of heterogeneous integration of electronics using additive manufacturing.

**Fig. 3:**
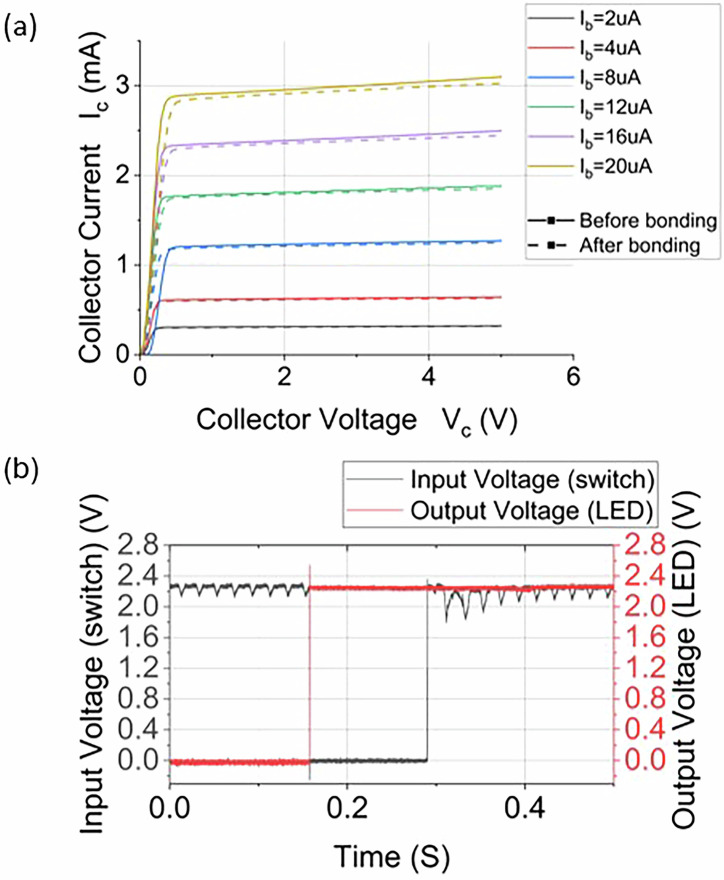
Biodegradable circuit characteristics. **a** The output characteristic of BJT before and after UPD bonding. **b** Input (switch) and output (LED) voltage of the Microcontroller circuit.

The first circuit is the Touch sensor switching circuit. The circuit was fabricated on PHBV substrates, featuring a packaged or unpackaged IC with a BJT array (SIS3045). Bare dies were used to minimise the need for non-degradable packaging, and as a consequence, the resulting PCBA consists of 99.9% recyclable or biodegradable materials. A switch array circuit, shown in Figs. 2a and 3c utilises a 5 V input voltage that, when the switch is open, applies voltage to the base of the BJT, keeping the BJT in an active state and shorting the connected LED to remain off. Conversely, closing the switch grounds the BJT, deactivates it and allows the LED to light up through the load resistor (R_L_). A touch sensor circuit employing a BJT array IC was also used, which transitions the BJT from a non-conducting to a conducting state, thus lighting an LED. This circuit is powered by a 9 V source (V_cc_), with operational dynamics visible in Fig. 3c. Figure 3d shows the Scanning Electron Microscope (SEM) image of the bonded bare die region highlighted in Fig. 3c, where three Ag wires printed using the UPD system connect the Ag tracks to the bonding pads on the bare die.

In assessing the IC’s performance with and without UPD printing, Fig. 3a presents a comparative analysis of the output characteristic curves obtained prior to bonding, while the dashed line illustrates the output characteristics post-UPD bonding. Reviewing output characteristic curves, there is a good overlap, indicating the UPD bonding is not significantly contributing to parasitic resistances. For example, at a collector voltage (V_c_) of 4 V and a base current (I_b_) of 20 μA, the collector current (I_c_) before bonding is recorded at 3.1 mA, compared to 3.04 mA after bonding—a subtle difference of 2%. This small discrepancy is due to the additional resistance from the UPD connections, but typically the same as the value experienced by wire bonding, indicating that UPD bonding has a minor impact upon the BJT’s performance at low frequencies.

The second circuit is the Binary counter circuit. Figure 2b, e illustrates a counter circuit using a packaged MSP430 microcontroller, which executes a pre-written programme to cycle through binary states displayed on a 2-bit LED, triggered by toggling a switch connected to pin 2.0. In this case, the substrate is PHBV, and after conductive track and IC recovery, 99.9% of the remaining materials are biodegradable. This circuit relies on input events and provides corresponding output signals, with Figures showcasing the LEDs in ‘11’ and ‘10’ binary states, respectively. Figure 2b utilises a 3.3 V power output from a computer’s USB port, demonstrating the circuits’ compatibility with low-voltage and portable power sources.

To analyse the performance of the MSP430 microcontroller circuit, a digital oscilloscope (PicoScope) was used to observe and record the waveforms from the input switch and the output LED, as illustrated in Fig. 3b. This evaluation focuses on the microcontroller’s stability and the reliability of the printed circuit. The analysis indicates that both input and output signals responded swiftly to the switch. Specifically, a sharp transition in the switch input and a corresponding rapid response in the LED output were noted, illustrating the microcontroller’s effective signal processing and control capabilities. Overall, the results demonstrate that circuits fabricated on biodegradable PHBV substrates *via* additive manufacturing techniques retain stable electrical performance, rapid signal response, and low noise levels, conclusively proving that environmentally sustainable electronics can achieve functional parity with conventional systems while eliminating ecological burdens.

Reliability is a critical requirement for future electronics made using DfR principles to go into deployable, commercial systems. Conventional PCBs are highly reliable owing to the use of solder masks to insulate Cu traces and protect the circuitry from oxidation, contamination and mechanical damage. Future work should focus on developing a solder mask that can be removed to enable Ag track recovery. However, reliability tests were conducted on samples at 65 °C (High temperature), 65 °C–85% (Damp heat) and shelf-life testing for 300 hours. All devices showed that track resistance remained within 10% of the original value. Bend tests were also conducted; the Ag tracks on PHBV were shown to survive more than 1000 bending cycles at 10 mm radius with no measurable change in resistance. Overall, we saw no variation over this test range, indicating short-term stability for reasonable normal operational conditions.

### Recycling trials

Recycling trials were conducted using aqueous FeCl_3_. Although HNO_3_is effective for the removal of the Ag, using FeCl_3_ (aq) provides a milder, lower impact, more selective alternative for Ag recovery and limits the intermixing of recovered Ag with organic residues^29^. Samples were measured by weight before and after printing and assembly. On average, 7.7 mg (±1.4 mg) is the increase in mass after printing the silver circuit onto the Fibernyl substrate. In order to determine the efficiency of recycling of the Ag tracks, the amount of Ag in the printed tracks had to be determined. Therefore, for Inductively Coupled Plasma Optical Emission Spectroscopy (ICP-OES) analysis, extraction was done using 35% HNO_3_ to convert the Ag on a stable substrate (glass) to water-soluble silver nitrate. There was some residue polymer binder present in the Ag ink, but Ag content in the conductive tracks was determined at 55.0 ± 6.7%.

**Fig. 4:**
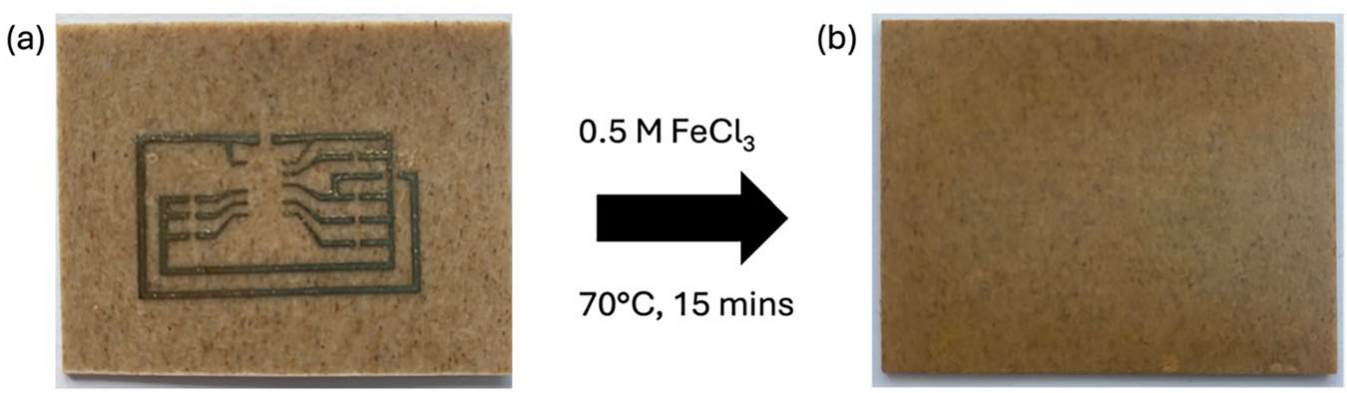
Printed circuit recycling. **a** Printed Ag as microcontroller circuit on Fibernyl substrate; **b** Fibernyl substrate after extraction of Ag tracks using 0.5 M FeCl_3_ (aq) solution.

Figure 4 shows that the Ag tracks are successfully removed following treatment with FeCl_3_(aq) solution. There are more detailed results shown in Supplementary Section ‘Circuit Recycling Detailed Result,’ and the demonstration in Supplementary Fig. 5. A faint outline of the circuit pattern can be noticed, but this appeared to be an intermixing layer between the ink binder and the substrate, and the substrate is to be reused; this can be easily removed by wiping with isopropanol. To confirm this, the recycled substrates were digested using 35% HNO_3_ (aq) (80 ml) at 80 °C for 1 h to extract any remaining silver. The resulting solutions were analysed by ICP-OES, with a concentration of 0.068 ppm determined, indicating clear Ag selective removal. This corresponds to an average remaining mass of 0.065 mg on the substrate after recycling. Based on the remaining mass of silver, 100% leaching according to the toxicity leaching characteristic procedure (TCLP) would result in an average concentration of 3.3 mg L^-1^, which is less than 5 mg L^−1^ (ref.^30^). The limit determined by the Resource Conservation and Recovery Act (RCRA)^31^, set by the United States Environmental Protection Agency. Therefore, after recycling, the substrate would be deemed acceptable for landfill disposal, reuse or recycling.

The extracted AgCl solid was converted to metallic Ag using the lye and glucose method, meaning the Ag content could be analysed by ICP-OES by being dissolved in nitric acid via conversion to silver nitrate. The ICP-OES results are shown in Table 2.

**Table 2:** Parameters to determine the extraction efficiency for recycling Ag tracks

Mass of printed track (mg)	7.7 (±1.4)
Mass of Ag in printed track (mg)^a^	4.3 (±0.8)
Extracted [Ag] (ppm) from digestion of substrate, measured by ICP-OES^b^	0.068 (±0.056)
Ag remaining on substrate (mg)^b^	0.065
Recycled [Ag] (ppm) measured by ICP-OES	384^c^, 3.64^d^, 3.64^d^
Mass of Ag recycled (mg)^e^	3.62 (±0.02)
Recycling efficiency (%)^f^	87.2 (±14.8)

^a^Mass of Ag determined by multiplying mass of printed track by 0.55, the fraction of Ag determined in printed tracks.

^b^Dilution factor of 1:9.41.

^c^Dilution factor of 1:10.

^d^Dilution factor of 1:1000.

^e^determined by scaling with mass of solution.

^f^(Mass of Ag extracted ÷ Mass of Ag in printed track) × 100).

An average recycling efficiency of 87% was determined across three samples, demonstrating the ability to effectively extract Ag from printed tracks for reuse. Given that the average mass of Ag in the printed tracks (4.3 mg) is very small, any small losses from the two filtration steps have a proportionally larger impact on the recycling efficiency, but this would be reduced when being carried out at a large-scale recycling process, and we estimate it would be >95%. Residual metals from the IC or other components (Cu, Si) were only present in trace quantities, and ICs retained performance after recovery, further confirmation of the selective nature of treatment with aqueous FeCl_3_. In conclusion, the recycling process demonstrated here offers a promising method for the removal and recovery of Ag from biodegradable substrates used in electronics, aligning with environmental sustainability goals while maintaining material value.

### Life cycle assessment

LCA was used to compare the environmental impacts between PCBs manufactured using the approach set out in this paper and those made from traditional FR-4. The environmental impact assessments cover the CML impact categories and cover the Abiotic Depletion (ADP), Acidification Potential (AP), Eutrophication potential (EP), Freshwater Aquatic Ecotoxicity Potential (FAETP), Global warming potential (GWP), Human Toxicity Potential (HTP), Ozone depletion potential (ODP), Photochemical Ozone Creation Potential (POCP) and Terrestrial ecotoxicity Potential (TETP) using the EcoInvent database^32^.

**Fig. 5:**
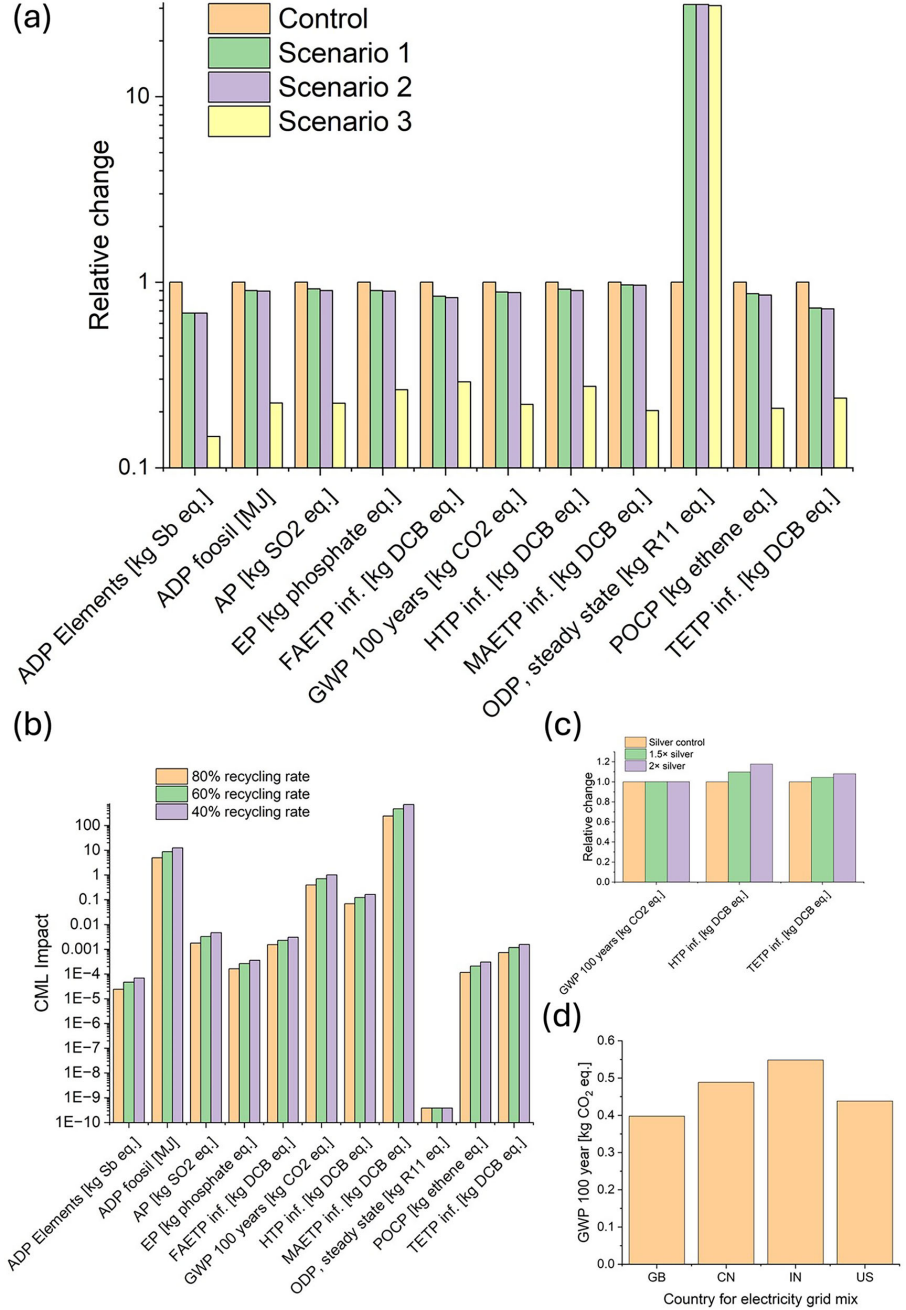
Biodegradable circuit LCA analysis. **a** Comparative LCA results for all CML impact categories for a (Control) FR-4 based PCB versus the PHBV based PCB approach for three different scenarios detailed in Table 3; **b** LCA comparison for different recycling rates; **c** Sensitivity analysis for selected impact categories for variation in silver thickness, and **d** Global warming potential for biodegradable PCB life cycle using energy mix from different countries.

LCA were made using the scenarios depicted in Table 3. In Table 4, the main materials and components used in all LCAs for fabrication usage are given, based upon the circuit demonstration in Fig. 2b and the results are shown in Fig. 5. First, we compared the impact of FR-4-based PCBS compared to PHBV analogues fabricated with additive manufacturing (Fig. 5a), described in the scenarios in Table 3. The baseline scenario with FR-4 substrates shows a higher environmental impact in several critical categories, such as GWP and HTP, largely due to the materials’ intensive energy processes and chemical treatments required during manufacturing and disposal. Switching to PHBV substrates significantly reduces these impacts. There is an increase in ODP, but it reaches only 3.8 × 10^-10^ kg trichlorofluoromethane (R-11) eq., a small impact. PHBV’s advantage stems from its biodegradable properties and lower energy requirements for processing. Furthermore, the source of the higher impact in FR-4-based PCBs is primarily due to the substantive process of etching copper and secondarily due to the substrate material differences. On the former, the quantity of Ag used is much lower than the Copper used in FR-4 production due to the use of additive printing. In addition, the FR-4 substrate consists of epoxy materials, glass fibres and BFR; all of which put a significant burden on the environment over their life cycle. As a result, in the scenario without recycling, PHBV already shows a reduced environmental load.

**Table 3:** Scenarios used for LCA of the FR-4 and PHBV-based circuits

Control	Circuit on FR-4, all components to landfill
Scenario 1	Circuit on PHBV all components to landfill
Scenario 2	Circuit on PHBV, recycled Ag and components (not IC)
Scenario 3	Circuit on PHBV, recycled Ag and IC

**Table 4:** Inventory of the components, energy consumption and materials used for the LCA scenarios

Component	Details	Quantity
Conventional	FR-4 etched copper	35 cm^2^–5 cm by 7 cm
PHBV circuits	PHBV sheet	2.65 g (5 cm by 7 cm)
	Ag ink	30 mg
Adhesives	Ag paste	2 mg
Components	1 MSP430 Microcontroller, 1 resistor, 2 LEDs	750 mg in total
Electricity	Circuit printing	0.01 kWh
	Circuit curing	0.033 kWh
	Use phase	0.01 kWh
	End of life (recycling)	0.025 kWh

We undertook a sensitivity analysis to determine the influence of variation on the most significant impact. This was carried out for Scenario 3 from Table 3. As shown in Fig. 5a, recycling, especially the IC, has the strongest effect on the environmental impacts. This was further illustrated in Fig. 5b, where the effect of different recycling rates was modelled, showing an increase in environmental impacts as the recovery rate decreases. The effect of variations in thickness in the deposited silver was modelled by varying the mass of silver in the LCA models (Fig. 5c). As the mass of silver used for the PHBV-based PCB is small, there is only a small increase in impact for most categories (e.g., GWP, shown in Fig. 5c). The largest increases in impact are in HTP and TETP, where silver use has a stronger influence. However, the generally small variation in impact with silver thickness highlights the advantage of additive manufacturing. Finally, the effect of electricity grid energy mix on the impact of PHBV PCB manufacture was modelled based on the country of electricity production (Fig. 5d). It is shown that the global warming potential can be increased by up to 38% based on different energy mixes used to generate electricity.

## Discussion

The results presented demonstrate a viable Design-for-Recycling (DfR) pathway for electronic manufacturing, integrating biodegradable substrates, fully additive manufacturing, and efficient, low-impact material recovery.

The selection of a suitable substrate is foundational to this approach. Our analysis of various biodegradable materials demonstrated that while options like Terranyl and Fibernyl suffer from high surface roughness and low thermal stability, materials such as P(3HB-co-4HB) and PHBV offer significantly lower roughness, which up to 50% less than FR-4 and sufficient thermal stability for curing processes up to 150 °C. This makes them the most suitable candidates for high-quality additive printing.

Building on this substrate choice, the additive manufacturing processes proved highly effective. The electrical resistivity of the printed Ag tracks (~1.4 ×10⁻⁴ Ω·cm), although higher than that of conventional etched copper, was demonstrated to be entirely sufficient for the functionality of low-power BJT and microcontroller circuits. Crucially, the use of UPD for wire bonding bare dies achieved a resistivity approaching that of bulk silver, offering conductivity comparable to traditional gold wire bonding but with lower material use and significantly reduced thermal resistance. The minimal impact on IC performance, a ~ 2% change in collector current, confirms that this DfR approach can achieve functional parity with conventional systems, demonstrating that environmentally sustainable electronics do not have to compromise on stable electrical performance and rapid signal response.

A key part of our DfR strategy is the EoL management. The recycling trials confirmed that an aqueous FeCl₃ solution provides a milder, lower-impact and more selective alternative to aggressive acids like HNO₃ for Ag recovery. The process demonstrated an 87% recovery efficiency at a lab scale, and post-treatment analysis confirmed that over 99% of the Ag was removed from the substrate. This renders the substrate acceptable for landfill disposal, reuse, or recycling under Resource Conservation and Recovery Act (RCRA) standards. This effective recovery method for a critical raw material is a significant step towards a circular economy for electronics. FeCl₃ is well-suited for use in the Ag recovery process due to its high chemical stability, regenerability, and well-established disposal pathway at the end of usage^33^. During Ag recovery, FeCl₃ is reduced to FeCl₂ while oxidising metallic Ag, and the resulting ferrous species can be readily re-oxidised to Fe³⁺ using electrolysis, for example, enabling multiple reagent reuse cycles. Loss of iron chloride is typically low, as Fe remains in solution and is not co-precipitated with Ag, while Ag recovery efficiencies are high due to the favourable and distinctive redox kinetics. Process effluents, consisting primarily of FeCl₃, can be treated using conventional neutralisation and precipitation steps to form stable iron hydroxides, which are non-hazardous and compatible with existing wastewater treatment, or as mentioned can be recycled back into upstream leaching^34,35^.

**Fig. 6:**
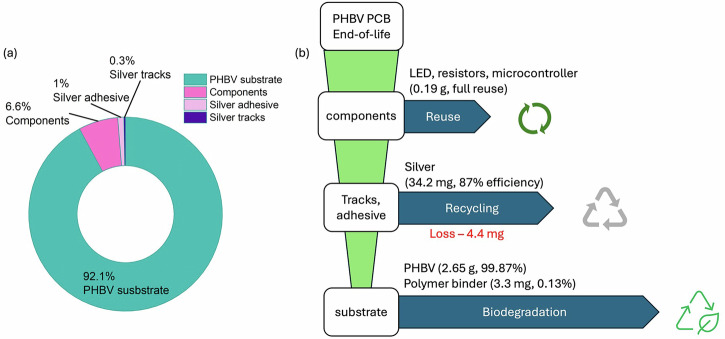
Material flow of PHBV PCB. **a** Breakdown of mass fraction of PHBV PCB, determined by a combination of mass measurements and ICP-OES analysis of silver extract; **b** Material flow of PHBV PCB at end-of-life.

The materials flow at end-of-life is shown in Fig. 6. The experimentally determined mass fractions of each material are shown in Fig. 6a, with the substrate accounting for the majority of the sample. The DfR approach allows for sustainable treatment at end-of-life, depicted in Fig. 6b, with components as we have shown that components can be reliably reused, silver can be recovered at high efficiency, and the remaining material is the substrate, which can be safely disposed of for natural degradation.

The Life Cycle Assessment (LCA) provides a comprehensive validation of this DfR approach, quantifying its environmental benefits. The switch from FR-4 to PHBV, combined with additive manufacturing and component recycling, resulted in a transformative environmental impact reduction of up to 80% across key categories like GWP and HTP. Comparative LCA of PHBV-based PCB scenarios 1–3 reveals distinct environmental trade-offs driven by component recovery strategies. Transitioning from Scenario 1 to 2 yields moderate reductions of around 5% across all LCA categories, primarily attributed to the recovery of Ag traces and passive components. However, the limited mass of recyclable passive components (190 mg total) coupled with energy-consuming DES processing partially offsets these gains. Scenario 3 demonstrates transformative improvements, achieving 60-80% lower environmental impacts than Scenario 1. This drastic reduction comes from the recovery of the microcontroller IC. IC recycling avoids the embedded impacts of the silicon fabrication process, which is responsible for 85% of semiconductor-related GWP due to high-purity material demands and energy-intensive cleanroom operations. Consequently, Scenario 3 outperforms conventional FR-4 systems(control) by 8–10 times in key metrics, with GWP reduced from 1.8 kg CO₂ eq (FR-4) to 0.4 kg CO₂ eq (PHBV). Overall, these LCA results validate that our approach of substitution of FR-4 with biodegradable PHBV substrates regarding environmental impact. Moreover, circular electronics require recovery strategies prioritising high-mass components. While passive device recycling marginally improves sustainability, IC recovery drives systemic impact reductions by addressing the semiconductor industry’s outsized environmental footprint.

In summary, WEEE poses significant environmental and sustainability challenges. It is therefore crucial to explore alternative manufacturing and recycling approaches that can maintain high performance while minimising environmental harm. In our work, we have demonstrated a fully 'design-for-recycling’ approach to produce two circuits using biodegradable substrate manufactured by additive printing, followed by UDP bonding of bare die chips. The combination of additive manufacturing, easy material recycling, and non-hazardous end products all contribute to an 80% reduction in the life cycle ecosystem damage potential relative to FR4-based devices, as validated by LCA.

This work proves the substantial potential benefits of a DfR approach in electronics. Looking ahead, several avenues for future work are identified to further advance this study.

- Firstly, long-term reliability testing of the circuits fabricated on biodegradable substrates, including the additive Ag tracks and UPD wire-bonding, under various environmental conditions (e.g., humidity, thermal cycling) is crucial to validate their suitability for real-world applications.
- Secondly, scaling up both the additive manufacturing processes and the FeCl₃-based recycling protocol beyond the laboratory setting is necessary to assess industrial feasibility and refine efficiency estimates.
- Thirdly, a comprehensive techno-economic analysis (TEA) should be conducted to evaluate the cost-competitiveness of this DfR manufacturing and recycling pathway compared to traditional PCB production and existing WEEE management streams.
- Further investigations should focus on assessing the functional yield and failure modes of recovered ICs, aiming to optimise the recovery process to significantly improve the rate of viable components for reuse, in parallel with developing biodegradable encapsulation methods.

In conclusion, this work demonstrates a fully integrated Design-for-Recycling electronics manufacturing process that combines biodegradable PHBV substrates, fully additive silver printing and ultra-precise deposition bonding to achieve high-performance, low-waste circuits. Key results show up to 99% material recoverability, laboratory-scale silver recovery efficiencies of ~87% (projected >95% at scale), and electrical performance comparable to conventional PCBs, with UPD Ag bonds approaching bulk silver resistivity. Two functional circuit demonstrators, including BJT arrays and a microcontroller system, validate stable operation with negligible performance penalty after additive integration. Life cycle assessment reveals up to a 90% reduction in environmental impact relative to FR-4 PCBs, with dramatic reductions in global warming and human toxicity potentials driven by additive manufacturing and component reuse.

## Methods

### Design for recycling approach

**Fig. 7:**
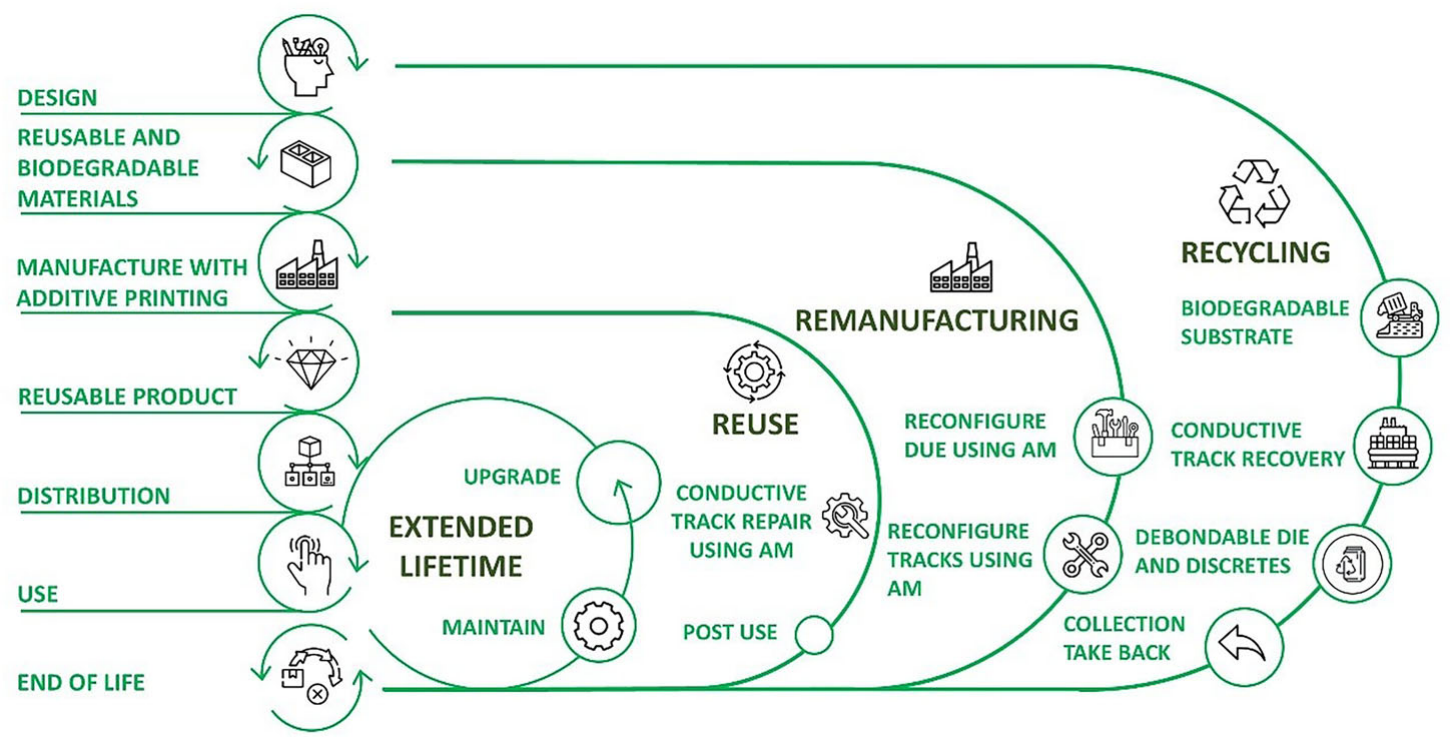
Block diagram for electronic manufacturing with the design for recycling. Block diagram in design, reusable and biodegradable materials, manufacturing with additive printing, reusable product, distribution, use and end-of-life process for electronic manufacturing.

This approach set out in this work fits into a general ‘Design for Recycling’ (DfR) framework by demonstrating how sustainable materials selection, low energy additive manufacturing and low-impact recycling processes can be used to create a closed-loop system for electronic production (as shown in Fig. 7). The work uses a framework of DfR principles by ensuring that each stage, including material selection all the way to end-of-life recovery is designed to minimise waste, material usage and maximise material recovery. For example, the use of biodegradable substrates with PHBV allows for either the reuse of substrate or degradation in natural environments, eliminating EoL waste. The adoption of additive Ag printing eliminates hazardous processing. Additive manufacturing also allows for reconfiguration and repair of conductive tracks using ‘drop-on-demand’ processing. Due to the relatively high value of Ag, it is economically feasible to recycle the conductive tracks at the end of life. ICs can be debonded and recovered for reuse or upgraded for better functionality. The use of FeCl₃-based recovery chemicals enables efficient reclamation of valuable metals with a low environmental burden^33^. This recovered Ag can be used for the remanufacturing of conductive tracks in PCBs. Overall, these various strategies provide a systems-level approach that supports circular PCB manufacturing and thus, aligns with a DfR framework for resource efficiency, lifecycle optimisation and circular electronics development.

### Substrate testing

Substrates were identified based on their temperature and solvent compatibility. To date, a robust comparison of biodegradable substrates for electronics has not been conducted. Substrates tested include polyhydroxyalkanoates (PHAs) such as poly(3-hydroxybutyrate-co-3-hydroxyvalerate) (PHBV)^36^. poly[(R)-3-hydroxybutyrate-co-4-hydroxybutyrate] (P(3HB-co-4HB))^37^. polyhydroxybutyrate (PHB) as well as poly(lactic acid) (PLA)^12^. which is compostable^38^. Additionally, this examination extends to paper-based substrates such as Terranyl and Fibernyl sheets as well as Hahnemühle Fine Art Baryta Satin 300, composed of 100% α-cellulose (hereafter referred to as ‘paper sheet’) and Hahnemühle Photo Rag Ultra Smooth 305, made from 100% cotton (hereafter referred to as ‘cotton sheet’). Comprehensive details regarding the properties and selection criteria of each material are available in the supplementary materials. Substrate attributes investigated include surface roughness and thermal stability, characterised by TGA (Discovery TGA5500-0553, TA Instruments) and differential scanning calorimetry DSC (DSC2A-01781, TA Instruments). Both TGA and DSC analyses were conducted under a nitrogen atmosphere. For DSC, a ramp rate of 10 °C/min was used with a nitrogen flow rate of 50 mL/min. For TGA, the samples were heated from room temperature to 600 °C at a rate of 10 °C/min. An Alicona G4 InfiniteFocus was used to measure the surface roughness of various biodegradable substrate materials.

### Conductive track deposition and assembly

The conductive track is printed by an Ag inkjet printer (BotFactory SV2). Initially, the surface of the PHBV was rinsed with DI water and solvents to ensure cleanliness. Effective surface preparation is critical to ensure ink adhesion for printed circuits, similar to protocols in semiconductor photolithography. However, conventional cleaning methods using water or solvent-based solutions are often incompatible with the unique material properties of biodegradable substrates. Therefore, tailored surface treatment procedures were developed for each material class.

For Cotton and Paper Substrates, due to their sensitivity to water and organic solvents, cotton and paper sheets were cleaned of particulate contamination using high-pressure, pure nitrogen gas. Subsequently, a dehydration bake was performed on a hotplate at 150 °C for 20 minutes. This step removes residual moisture without compromising the structural integrity of the fibrous substrates, preparing them for subsequent printing.

For Commercial Bioplastics (Terranyl and Fibernyl), the surfaces of Terranyl and Fibernyl sheets, being relatively water-resistant, were gently wiped with a cloth dampened with isopropyl alcohol (IPA) to remove surface impurities; soaking was found to be unnecessary. Following the wipe-down, the sheets were heated at 120 °C for 20 min to devolatilize residual solvents and moisture. This temperature was selected to prevent thermal damage, as these materials exhibit lower heat tolerance than the paper-based substrates.

For Other Polymeric Substrates (P(3HB-co-4HB), PHA, PLA, PHB, PHBV), a preliminary flattening step was often required. This was achieved by pressing non-planar substrates between two smooth glass or metal plates on a hotplate at 120 °C. Once planar, these materials were cleaned using the same IPA wipe-down procedure as Terranyl and Fibernyl. The heating temperature and duration for both flattening and post-cleaning dehydration were optimised for each specific polymer to ensure flatness and moisture removal without inducing thermal degradation.

Curing was conducted at the lowest possible temperature that still ensured complete ink drying, which was optimised at 120 °C. To evaluate the integrity of these tracks, continuity and conductivity tests were performed, characterising the material properties and the effectiveness of the printing process. After printing the conductive tracks, a bare die was embedded into the substrates. The bond pads of the die were connected to the conductive tracks using the XTPL^TM^ Delta Printing System Ultraprecision Dispense (UPD) printer to avoid the use of non-degradable epoxy, which is used in conventional ICs. Overmoulding with biodegradable polymers can be conducted to ensure mechanical and environmental stability. The UPD system deposits extremely fine lines of Ag tracks down to 2 μm in width^39^. The bond pad size on our bare die is 100 × 100 μm with 170 μm pitch, meaning that the UPD system can write bond lines with sufficient resolution. The UPD’s printed tracks also require a curing process, which was also limited to 120 °C. Subsequently, the remaining components (SMD resistors, LEDs, etc.) are mounted on the circuit board using Ag conductive adhesive substrates.

### Recycling trials

To demonstrate circularity, recycling trials were conducted. Brines with redox catalysts have been shown to be effective at selective recovery of a single metal while reducing the toxicity of the process^40–42^. In our work, we selected a redox catalyst based on FeCl_3_ as it can significantly reduce the environmental impact of silver recovery when compared to aggressive acids such as HNO_3_. The metallic silver is oxidised by the FeCl_3(aq)_ solution to form AgCl (Eq. 1), although anionic silver chloride species can also be formed^43^. AgCl is easily recovered by filtration and was converted to metallic Ag using sodium hydroxide and glucose, where AgCl is converted to Ag_2_O and then reduced back to metallic Ag.

1 $${Ag}+{{FeCl}}_{3}\to {AgCl}+{{FeCl}}_{2}$$

To determine the efficiency of recycling, the mass of silver printed had to be determined. Some substrates, e.g., Fibernyl, have water content, and the mass of substrate therefore decreases during annealing if the substrate has not been pre-dried. Therefore, substrates were dried prior to printing with Ag and weighed. After printing Ag tracks, the substrates were weighed again to determine the mass printed onto the substrate. For material recovery, the coated substrates were stirred in a 0.5 M FeCl_3_ aqueous solution at 70 °C for 15 min before the substrates were removed, washed with deionized water and dried again to remove any absorbed water. The resulting precipitate in solution was collected by filtration, washed with deionised water and dried. The substrates were re-weighed to determine the mass extracted. The filtered precipitate was treated with aqueous sodium hydroxide solution (0.54 M) and glucose (1.15 molar equivalents) and heated at 80 °C for 15 minutes. The light brown solution was filtered, and the resulting solid was washed with deionized H_2_O and acetone. For ICP-OES analysis, solutions were prepared using the extracted Ag by dissolving the solid in 35% HNO_3_(aq) (1 ml) and diluted using deionized H_2_O (9 ml). These solutions were further diluted with deionized H_2_O (20× dilution) for ICP-OES measurements. The ICP-OES measurements were carried out using an Agilent 5900 system.

### Life cycle assessment goal and scope

This study conducts a comparative LCA to evaluate the environmental impacts of 15 cm² FR-4 and PHBV-based PCBs across their cradle-to-grave life cycles. The analysis focuses on differing substrate materials, conductive track fabrication methods, and EoL management strategies. System boundaries encompass material extraction, manufacturing, and EoL processing, with usage phase impacts excluded due to identical low-power circuitry assumptions and reliability exceeding typical IoT device lifespans. The FR-4 baseline model builds upon prior methodologies from Grant et al.^16^ and Ozkan et al*.*^25^, implemented using Sphera’s GaBi software. For FR-4 PCB EoL, a landfill disposal scenario was modelled, reflecting current WEEE management practices, as most electronics are still landfilled. In contrast, three EoL scenarios were evaluated for PHBV PCBs: (1) Full landfill disposal; (2) Component reuse (excluding microcontrollers) with substrate landfilling; (3) Full component reuse with substrate landfilling. Scenario 2 addresses the prevalent obsolescence of microcontrollers in discarded electronics, where rapid technological advancements render them outdated, while passive components (e.g., capacitors, resistors, inductors) often retain functional viability for reuse in new devices. Scenarios 2–3 assume 90% component recovery efficiency and complete metal reclamation from conductive tracks, with system boundaries encompassing material liberation, separation, and refining. The LCA was regionally specified using data from Great Britain (GB). Life cycle inventories were constructed using GaBi’s elemental flow taxonomy, categorising inputs as elementary flows, waste streams, or tracked non-elementary processes. Ecoinvent datasets informed EoL treatment modelling, with substitution accounting applied to quantify environmental credits from material recycling.

## Supplementary information

Supplementary Materials_npj_mat_sus_rev_0218

## Data Availability

The datasets generated and/or analysed during the current study are not publicly available as the data require controlled access to ensure appropriate usage and interpretation, but are available from the corresponding author on reasonable request.
