# Supplementary material for: Design for recycling in electronic manufacturing: enabling circularity and lower impact manufacturing through heterogeneous integration and lower impact recovery
Source: Npj Mater Sustain. 2026 Mar 16;4(1):10. doi: 10.1038/s44296-026-00098-8 (PMC12992108; doi:10.1038/s44296-026-00098-8)
Supplement: Supplementary file 1 — Supplementary Materials_npj_mat_sus_rev_0218 [file 44296_2026_98_MOESM1_ESM.pdf]

# Supplementary Materials

**Design for Recycling in Electronic manufacturing: enabling circularity and lower impact manufacturing through heterogenous integration and lower impact recovery**

**Tianwei Zhang[1,*], Jonathon Harwell[1], Joseph Cameron[1], Shoushou Zhang[1,2], Hadi Heidari[1], Jeff Kettle[1]**

**[1] James Watt School of Engineering, University of Glasgow, Glasgow, G12 8QQ, UK**

**[2] Bangor College, Central South University of Forestry and Technology, Changsha, China**

**[*] Tianwei.Zhang@glasgow.ac.uk**

## Basic Information and Results of Biodegradable Substrates

A selection of commercially available, high-fidelity art papers was evaluated as potential biodegradable substrates for printed circuit board (PCB) applications. Specifically, Hahnemühle Fine Art Baryta Satin 300 and Photo Rag Ultra Smooth 305 were chosen for their sustainable origins and absence of optical brighteners[1] . The Fine Art Baryta Satin 300 is an α-cellulose-based paper with a satin-gloss inkjet coating that ensures high-resolution printing, a critical requirement for circuit layouts [2] . The Photo Rag Ultra Smooth 305, a 100% cotton paper, features an exceptionally smooth, matte-coated surface optimized for fine detail and color depth [1] . As illustrated in Supplementary Figure 1, both papers possess textures suitable for high-quality printing and required only cutting to the desired dimensions prior to use.

Commercially produced biodegradable bioplastics were also investigated. These included

Terranyl®, a compostable sheet material derived from potato starch residue [3] , and Fibernyl, a composite sheet made from Elephant Grass (Miscanthus giganteus) [4] . Terranyl® is certified for non-industrial composting and exhibits properties comparable to Polystyrene, making it suitable for high-precision applications. Fibernyl is notable for its high $CO_2$ sequestration rate and natural texture. Both materials, shown in Supplementary Figure 1, were supplied as rigid sheets and were readily cut to size for subsequent PCB fabrication.

Three distinct polyhydroxyalkanoate (PHA) bioplastics—as poly(3-hydroxybutyrate-co-3-hydroxyvalerate) (PHBV), poly[(R)-3-hydroxybutyrate-co-4-hydroxybutyrate] (P(3HB-co-4HB)) , polyhydroxybutyrate (PHB)—were procured as pre-manufactured sheets or rolls. PHBV, supplied as a flexible roll, was thermally flattened at approximately 100℃ to create smooth, planar sheets suitable for printing. The P(3HB-co-4HB) is produced via a mold-pressing process that incorporates an alcoholysis step to enhance its ductility relative to the more brittle PHB. This enhanced flexibility makes P(3HB-co-4HB) and PHBV more robust against cracking during handling and processing. From a thermal perspective, these PHB-type polymers are promising due to high melting temperatures (approx. 170-180°C) suitable for soldering, but their very low glass transition temperatures (typically below 10°C) present a significant challenge for maintaining mechanical stability at elevated operating temperatures. Both P(3HB-co-4HB) and PHB were supplied as rigid plates directly from moulding.

In addition to commercial substrates, we fabricated sheets of polylactic acid (PLA), polyhydroxyalkanoate (PHA), and polybutylene adipate terephthalate (PBAT) in-house. PLA

sheets were produced using fused filament fabrication (3D printing), enabling customization of substrate dimensions, though feature precision was inherently limited by the filament diameter. Thermally, PLA offers an inverse profile to the PHAs; its low melting point (approx. 10°C lower than PHAs) makes it fundamentally unsuitable for standard PCB soldering processes. In contrast, PHA and PBAT sheets were created by melt-casting granular polymer at 150-200℃ in flat mould. The absence of a vacuum-equipped moulding setup resulted in occasional surface porosity and bubbles in the cast sheets. Consequently, for printing evaluations, only defect-free areas of these sheets were carefully selected and measured.

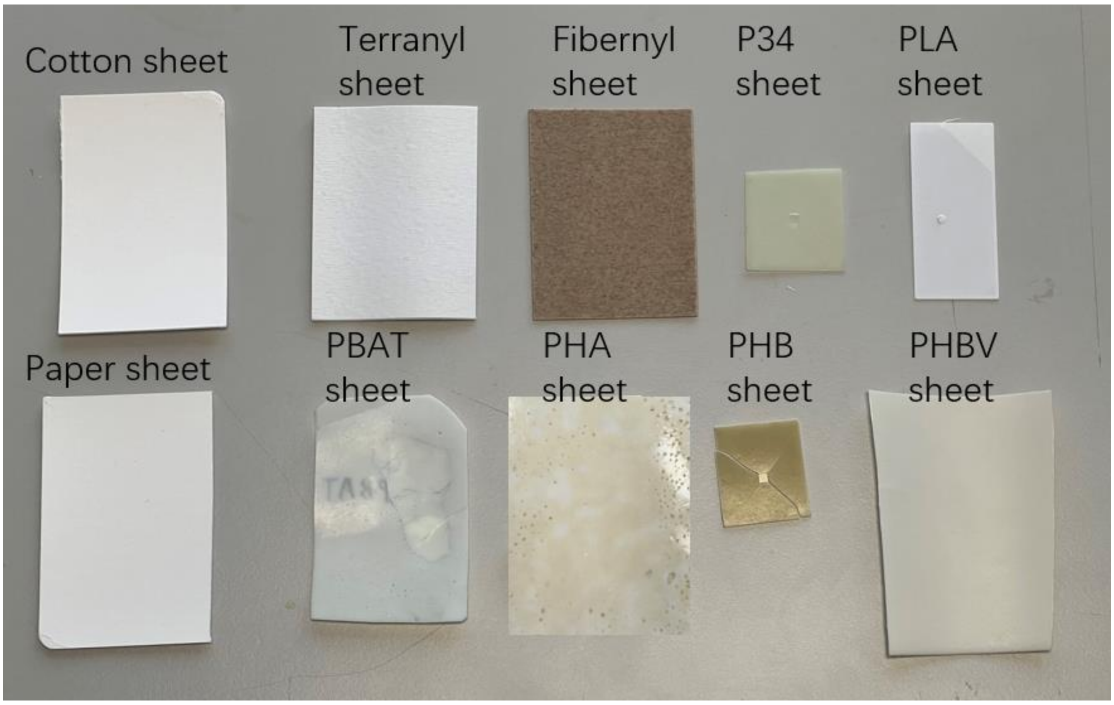


**Supplementary Figure 1**. Biodegradable substrates used in this paper

**Supplementary Table 1**. Temperature of 5% mass loss in TGA measurement, melting point, glass transition temperature of selected biodegradable substrate

| Substrate | Fibernyl | Terranyl | P(3HB-co-4HB) | PBAT | PHA | PHB | PHBV | PLA |
|---|---|---|---|---|---|---|---|---|
| Temperature of 5% mass loss (TGA) (℃) | 285 | 278 | 253 | 359 | 260 | 255 | 243 | 330 |
| Melting point (℃) | 155 | 150 | 180 | 120 | 175 | 175 | 172 | 165 |

## Die Embedding Results

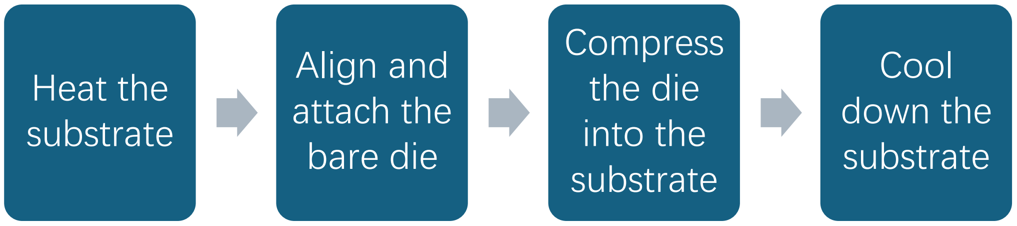


**Supplementary Figure 2**. Flowchart of die embedding process

Bare die embedding is an innovative process for creating a planar surface topology on a PCB in this paper. This planarity is critical for eliminating the Z-axis step height (typically ~50 µm) between the die surface and the substrate's conductive tracks, which simplifies subsequent

interconnection steps such as wire bonding [5] [6] . As outlined in Supplementary Figure 2, we developed a thermo-compression embedding process that leverages the thermoplastic nature of certain biodegradable polymers. This method is unachievable with rigid thermoset substrates like FR-4, which cannot be thermally reshaped to integrate components.

The process was demonstrated using PHBV, a flexible biopolymer with a softening point of approximately 140℃. Firstly, the PHBV substrate was heated to its softening point on a hotplate. A bare die was then aligned with pre-fabricated Ag conductive tracks (shown in section 2.3 in the main paper) and placed onto the softened surface. Subsequently, a smooth glass or metal plate was positioned over the die, and controlled pressure was applied to embed the component until its surface was flush with the polymer substrate. The assembly was allowed to cool to room temperature under continuous pressure to prevent die displacement as the polymer solidified. Upon removal of the plate, the die remained securely fixed in a planar alignment with the substrate surface, as shown in Supplementary Figure 3.

The feasibility of this thermo-compression technique is highly dependent on the substrate's thermal and mechanical properties. Materials lacking a distinct softening transition before degradation, such as the cotton and paper substrates, are incompatible with this embedding method. Conversely, for other compatible polymers, the process parameters required optimization. For instance, PHB, which is significantly more brittle than PHBV, necessitated precise temperature control to achieve sufficient malleability for embedding without causing fractures under pressure.

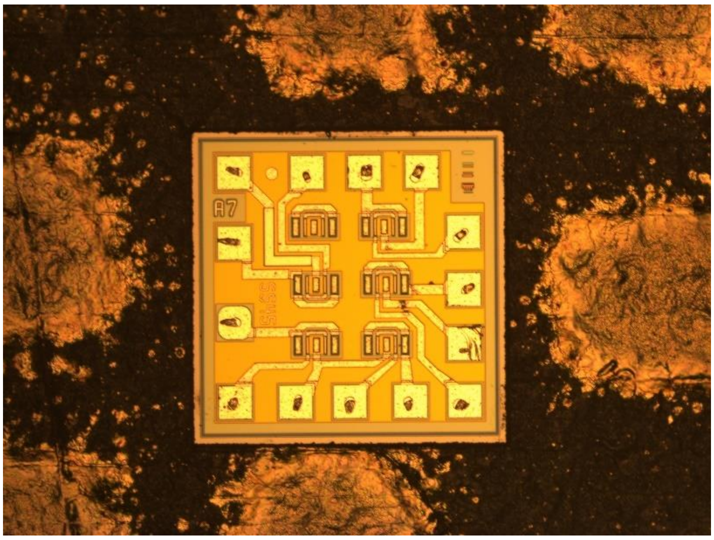


**Supplementary Figure 3**. PHBV sheet with embedded BJT die surrounded by Ag conductive tracks

## Packaged/SMD Device Mounting Results

While ultra-precision dispensing (UPD) is ideal for printing fine conductive tracks, it is less suitable for mounting larger packaged chips or surface-mounted devices (SMDs) for both technical and practical reasons. From a practical standpoint, the UPD process is known for its high material costs due to the special requirement for its very fine ink, and it is very time-consuming because of its micrometre-scale nozzle diameter. Technically, the fine printed structures lack the mechanical robustness required to support heavier components, creating a risk of bond failure under the mechanical and thermal stresses encountered during operation.

Therefore, an Ag-based conductive paste[7] was selected as the mounting adhesive, offering three key advantages. First, using a homogenous Ag-based system for both tracks and connections eliminates material interfaces, mitigating the risk of electrochemical corrosion

and simplifying end-of-life recycling. Second, the paste provides a strong mechanical bond capable of supporting the SMD components. Finally, the chosen paste cures at room temperature, a critical requirement to avoid thermally induced damage or deformation of the biodegradable substrates.

Supplementary Figure 4 shows an SMD LED mounted using this method. The Ag paste serves a dual function: it establishes a reliable electrical connection and provides robust mechanical adhesion. The paste effectively fills the vertical gap created by the component's thickness (approx. 0.28 mm) [8] , ensuring a stable and planar final assembly.

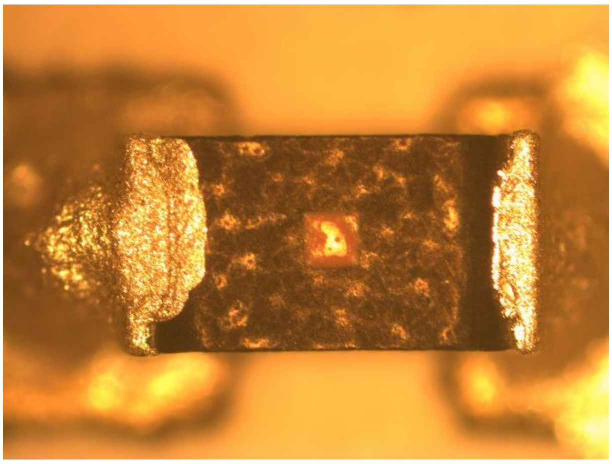

**Supplementary Figure 4**. SMD LED attached to substrate by Ag epoxy

## XTPL Resistivity Measurement Results

The measured resistance of this reference wire was 0.5 ohms. Given the short length of the bonding wires, typically less than 0.1 cm, the resistance contributed by the XTPL printed wires to the overall circuit can be estimated to not exceed 0.1 ohms. This minimal resistance confirms that the presence of XTPL printed wires does not adversely affect the circuit performance.

To calculate the resistivity of the Ag used in the XTPL process, the following formula for resistivity ($\rho$) is employed:

$$\rho = R \times \frac{A}{L} \quad (1)$$

Where R is the resistance, A is the cross-sectional area, and L is the length of the wire. Assuming the diameter of the wire is 20 microns, the cross-sectional area A can be calculated as:

$$A = \pi \left( \frac{20 \times 10^{-6} \text{ meters}}{2} \right)^2 = 3.14 \times 10^{-10} \text{ m}^2 \quad (2)$$

Given $R$=0.5 ohms and L=0.5 cm, the resistivity ($\rho$) is: $3.14\times10^{-6}$ Ω·cm.

## Circuit Recycling Detailed Results

Supplementary Figure 5 provides a visual validation of the substrate's recyclability following the complete removal of the printed Ag track. The experiment demonstrates a two-stage cleaning process designed to restore the Fibernyl substrate to a state suitable for reuse.

Initially, after the chemical extraction of the Ag using a ferric chloride ($FeCl_3$) solution, a faint residual pattern remains visible on the substrate surface, as depicted in Supplementary Figure 5a. The magnified inset shows this is not particulate AgCl but rather a faint mark corresponding to the original circuit layout. This is attributed to a thin, adsorbed layer of the ink's binder, which has been attached to the substrate during the printing and curing process. While the conductive material has been fully stripped, this minor organic residue persists through the initial chemical wash.

The subsequent cleaning step, shown in Supplementary Figure 5b, simply involves a wipe with isopropanol. This treatment is highly effective in dissolving and removing the residual binder. As confirmed by the magnified view, the pattern is completely removed, leaving no visible trace of the printed circuit. The substrate's surface is restored to a clean and homogenous state. This straightforward and effective recycling protocol underscores the reusability of the Fibernyl material, allowing for multiple cycles of circuit fabrication without significant degradation or complex reprocessing.

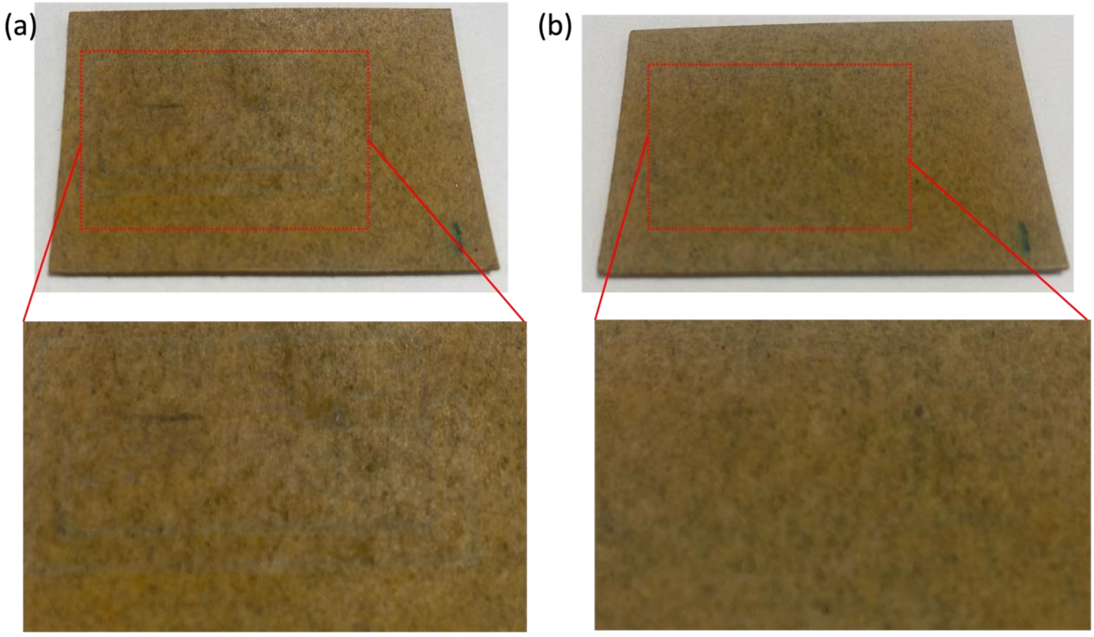


**Supplementary Figure 5**. Photographs of Fibernyl substrates after extraction of Ag track with 0.5 M $FeCl_3$(aq) solution before (a) and after wiping with isopropanol (b). The outline of the printed circuit is visible in the substrate after extraction and washing. but this is removed with simple treatment of wiping with isopropanol.

# Reference


[1] Fotospeed. *Hahnemühle Photo Rag Ultra Smooth 305*. at https://fotospeed.com/photo-rag-ultra-smooth-305.html (2024).

[2] Fotospeed. *Hahnemühle Fine Art Baryta Satin 300*. at https://fotospeed.com/fine-art-baryta-satin-300.html (2024).

[3] Bioplasticshop.com. *Terranyl® Sheet White*. at https://bioplasticshop.com/products/terranyl-en/terranyl-sheet-white1000x600x1mm/ (2024).

[4] Bioplasticshop.com. *Fibernyl® Sheet Black*. at https://bioplasticshop.com/products/fibernyl-en/fibernyl-sheet-black-1000x600x2mm-2/ (2024).

[5] Silicon Supplies. *SiS3045 - Wafer / Die Information*. at https://siliconsupplies.com/media/literature/SS-Obsolete/SiS3045.pdf (2016).

[6] Die Devices. *MSP430G2252*. at https://diedevices.com/product-detail/Microcontroller/16-bit/Ultra_Low_Power/MSP430G2252 (2025).

[7] RS PRO. *RS PRO Silver Conductive Paint, 20 g Bottle*. at https://docs.rs-online.com/cd63/A700000008879603.pdf (2025).

[8] RS PRO. *RS PRO White SMD LED, 1206 Package*. at https://docs.rs-online.com/21eb/0900766b8157cb45.pdf (2025).
